# Metaplastic Carcinoma of the Breast with Neuroectodermal Stromal Component

**DOI:** 10.4061/2011/191274

**Published:** 2011-02-09

**Authors:** Tibor Tot, Juan José Badani De La Parra, Leif Bergkvist

**Affiliations:** ^1^Department of Pathology and Clinical Cytology, Central Hospital, S-791 82 Falun, Sweden; ^2^Department of Pathology, Central Hospital, S-721 89 Västerås, Sweden; ^3^Department of Surgery and Center for Clinical Research, Uppsala University, Central Hospital, S-721 89 Västerås, Sweden

## Abstract

A unique case of metaplastic breast carcinoma with an epithelial component showing tumoral necrosis and neuroectodermal stromal component is described. The tumor grew rapidly and measured 9 cm at the time of diagnosis. No lymph node metastases were present. The disease progressed rapidly and the patient died two years after the diagnosis from a hemorrhage caused by brain metastases. The morphology and phenotype of the tumor are described in detail and the differential diagnostic options are discussed.

## 1. Introduction

Metaplastic breast carcinoma is a rare tumor and represents a heterogeneous group of lesions. According to the World Health Organization (WHO) classification, these lesions are divided into purely epithelial and mixed epithelial—mesenchymal tumors, with the latter called carcinosarcoma when the mesenchymal component is malignant [1, 2]. The mesenchymal component of carcinosarcomas most often exhibit chondroid, osteoid, or rhabdoid differentiation, and neuroid differentiation of this component is extremely rare. In this paper, we present a unique case of metaplastic breast carcinoma with an epithelial component showing tumoral necrosis and neuroectodermal mesenchymal component. 

## 2. Clinical History

The patient was a 53-year-old Kurdish woman with a large, rapidly growing mass in her right breast, but who was otherwise healthy. The patient had no family history of breast cancer, had given birth to seven children, and was still menstruating. At the time of presentation, the patient had a hard 9 cm tumor in her right breast that was visible on inspection. There were no palpable nodes in the axilla. Mammography showed a multinodular tumor, the largest nodule being 65 mm, with calcifications. Ultrasound confirmed the presence of a well circumscribed nodular tumor with mixed echogenicity and revealed enlarged pathological nodes in the axilla. A preoperative core needle biopsy of the breast lesion showed structures of an obviously malignant small cell tumor with a high proliferation index, and it was negative for estrogen and progesterone receptors and HER-2. preoperative neoadjuvant chemotherapy was given: three cycles of epirubicin and docetaxel, followed by three cycles of docetaxel. However, no substantial tumor remission was observed, and after five cycles a mastectomy and axillary lymph node dissection was performed. Because of the poor response to neoadjuvant chemotherapy, it was decided to give four cycles of carboplatin and gemzitabin (after a negative bone scan and computed tomography of the thorax and abdomen) followed by radiotherapy to the chest wall. Five months after the termination of radiotherapy dissemination was diagnosed to the liver, adrenal glands, and lungs. Despite new chemotherapy, first with a fluorouracil, epirubicin, and cyclophosphamide combination, and thereafter a combination of carboplatin and paclitaxel together with bevacizumab there was a rapid progress and the patient died two years after diagnosis from a haemorrhage caused by brain metastases. 

## 3. Materials and Methods

Histological analysis was performed on formalin-fixed, paraffin-embedded tissue. The immunohistochemical reactions were carried out using Dako autostainer. The following primary antibodies were used: estrogen receptor (Novocastra, clone 6F11, 1 : 100), progesterone receptor (Novocastra, clone 16, 1 : 100), c-erbB-2 oncoprotein (Dako, HER-2), mammoglobin (Dako, clone 304-1A5, 1 : 1), CD56 (Zymed Laboratories, clone 123C3, 1 : 100), CD99 (Dako, clone 12E7, 1 : 100), NSE (Dako, clone BBS/NC/VIH14, 1 : 500), synaptophysin (Novocastra, clone 27G12, 1 : 150), chromogranin A (Dako, clone DAK-A3, 1 : 3000), high molecular weight cytokeratin (CK; Dako, clone 34BE12, 1 : 150), CK8/18 (Novocastra, clone 5D3, 1 : 50), CK20 (Dako, clone Ks20.8, 1 : 100), CK7 (Dako, clone OVTL 12/30, 1 : 200), e-cadherin (Dako, clone NCH-38, 1 : 50), vimentin (Dako, clone Vim3B4, 1 : 600), calponin (Dako, clone CALP1, 1 : 600), p63 (Biocare Medical, clone BC4A4, 1 : 100), CD10 (Dako, clone 56C6, 1 : 50), and S-100 protein (Dako, clone ZO311, 1 : 3000). EWS-rearrangement for EWS/FLI typ 0.5 and EWS-ERG was tested using real-time PCR analysis. 

## 4. Pathological Features

**Figure 1:**
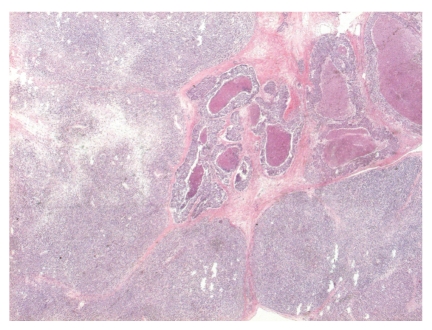
Scanning magnification of the tumor with the epithelial component with tumoral necrosis in the centrum. H&E stain.

**Figure 2:**
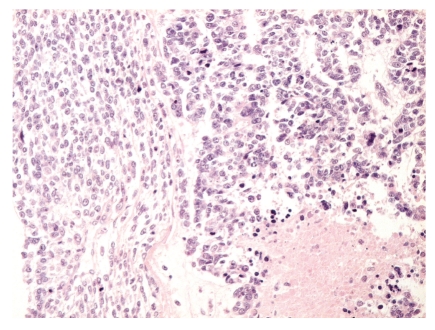
High-power magnification of the interface area between the epithelial and the mesenchymal components of the tumor.

The tumor appeared as a 90 mm, whitish mass upon macroscopic examination. The axillary portion of the specimen contained ten lymph nodes. Microscopically, the tumor showed solid areas of small cells and large necrotic areas. In the central part of the tumor, typical “comedo-like” carcinoma structures (structures with tumoral necrosis) were found that comprised roughly 10% of the tumor mass (Figure 1). These duct-like structures were composed of cohesive medium-size atypical cells and had necrotic debris in the lumen. No myoepithelium was seen around the structures. The small, relatively monomorphous cells that comprised roughly 90% of the tumor had scanty cytoplasm, exhibited a focally perivascular distribution, and sometimes appeared in cell files, but were most often arranged in large solid areas (Figure 2). No rosette-like structures were seen. Importantly, the entire cell population of the mesenchymal component demonstrated identical morphology; no signs of chondroid, osteoid, rhabdoid, or other differentiation were observed.

**Figure 3:**
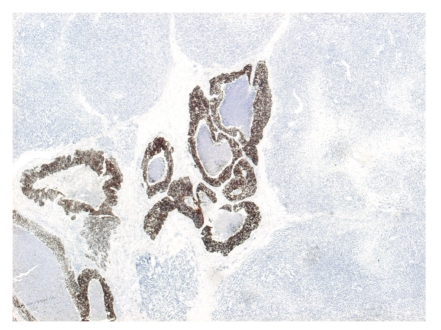
Detail from Figure 1 stained for high molecular weight cytokeratins.

**Figure 4:**
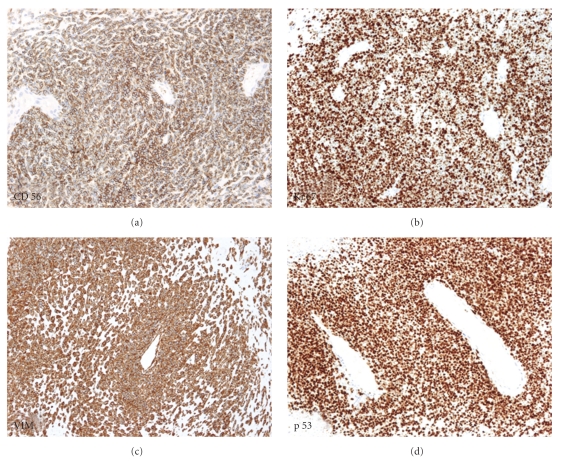
Combined image demonstrating the immunophenotype of the mesenchymal component of the tumor showing intensive diffuse CD56 positivity (a), vimentin positivity (c), high Ki-67 index (b), and p53 expression (d).

The structures with tumoral necrosis exhibited a clear epithelial phenotype and expressed high molecular weight and low molecular weight cytokeratins (Figure 3) and e-cadherin, and they were negative for vimentin. These structures weakly and focally expressed CD56. No in situ component could be demonstrated in the epithelial part of the tumor using the myoepithelial markers calponin, p63, and CD10. The solid component of the tumor exhibited a mesenchymal phenotype: no reaction with cytokeratins but strong diffuse vimentin positivity. The mesenchymal component of the tumor strongly and diffusely expressed the neuroid markers NSE and CD56 (Figure 4). No positivity for the estrogen and progesterone receptors, HER-2, mammoglobin, calponin, p63, chromogranin A, or synaptophysin was detected in any part of the tumor. CD10 stained both components of the tumor focally in about 10% of the section surfices. S-100 protein was present in only the dendritic cells in the stroma of the tumor. CD99 showed unspecific focal cytoplasmic reaction in the stromal component. The RT-PCR analysis for EWS/FLI1 and EWS/ERG turned out negative. The tumor cells in both the epithelial and mesenchymal portion of the tumor exhibited a very high percentage of p53-positive cells. The Ki-67 proliferation index was as high as 90% (Figure 4). No axillary lymph node metastases were present, but some of the nodes contained benign nevus structures in their capsule. 

Based on the morphology and immunophenotype of the tumor, the diagnosis of metaplastic carcinoma with a neuroectodermal stromal component was made. 

## 5. Differential Diagnosis

The presence of epithelial and mesenchymal elements in the same tumor is the hallmark of metaplastic breast carcinomas with a heterologous component, also named carcinosarcomas. In our case, the structures with tumoral necrosis clearly exhibited an epithelial morphology and immunophenotype, in contrast to the mesenchymal component expressing vimentin and the neuronal markers CD56 and NSE. The entire tumor showed signs of malignancy, such as rapid growth, extremely high proliferation index, and diffuse expression of p53. All of these findings motivated the diagnosis of metaplastic carcinoma with a neuroectodermal mesenchymal component, which represents a real rarity; no corresponding published case could be found in the English literature. 

Primitive neuroectodermal tumor (PNET) was one of the main differential diagnostic alternatives in our case. Four cases of primary breast PNET has been reported in the literature [3], but all lacked any epithelial component. PNET may express keratins in some cases, but the biphasic (epithelial-mesenchymal) character is not a feature of this tumor [4]. The stromal component in our case did not express CD99 and did not show the typical molecular genetic patterns which are the hallmarks of PNET.

The case of metaplastic breast carcinoma with neuroglial differentiation published by Golshan et al. [5] is similar to our case, but no glial differentiation was seen in our tumor. In addition, the diagnosis in the previous case was seriously questioned by Hameed and Dehner [6] because the tumor lacked a typical epithelial component. Milanezi et al. [7] published a series of breast carcinomas, among them were five metaplastic carcinomas, stained for CD99. Four of the metaplastic tumors expressed CD99, but only one of them expressed it in the stromal component. 

The presence of a heterologous component differentiated our metaplastic carcinoma from a purely epithelial neuroendocrine carcinoma of the breast. The cells of these tumors express epithelial and endocrine markers at the same time. In the literature, we found a single case of “primary small cell carcinoma” of the breast that did not express epithelial markers at all, but expressed CD56 and NSE [8]. This tumor was very similar to the mesenchymal component of the tumor in our case, but it was not biphasic and expressed synaptophysin, unlike our case.

The focal distribution of the invasive component indicates the need for thorough sampling of such a large tumor for proper diagnosis. The relatively monotonous stromal neuroectodermal component may be misdiagnosed as neuroendocrine carcinoma or PNET in absence of thorough sampling and adequate immunohistochemical support. 

Lastly, the metastatic character of the tumor was also considered as a differential diagnostic option; however, an extensive clinical and radiological search did not detect any additional tumor focus in our patient. The histological appearance of the typical comedo-like epithelial component also favored the primary localization of the tumor in the breast, though an in situ component could not be verified. 

## 6. Conclusions

The biphasic character of the lesion, obvious signs of malignancy, and expression of neuroectodermal markers in the stromal component qualifies the reported case for being metaplastic carcinoma with neuroectodermal stromal component. Up to our knowledge, this is the first such case reported in the literature.
